# Antioxidant network-based signatures cluster glioblastoma into distinct redox-resistant phenotypes

**DOI:** 10.3389/fimmu.2024.1342977

**Published:** 2024-04-18

**Authors:** Yihan Yang, Sanket More, Frederik De Smet, Steven De Vleeschouwer, Patrizia Agostinis

**Affiliations:** ^1^ Research Group Experimental Neurosurgery and Neuroanatomy, Department of Neurosciences, KU Leuven, Leuven, Belgium; ^2^ Laboratory of Cell Death Research & Therapy, Department of Cellular and Molecular Medicine, KU Leuven, Leuven, Belgium; ^3^ Vlaams Instituut voor Biotechnologie (VIB) Center for Cancer Biology Research, Leuven, Belgium; ^4^ Department of Imaging and Pathology, KU Leuven, Leuven, Belgium; ^5^ Leuven Institute for Single-Cell Omics (LISCO), Leuven, Belgium; ^6^ Department of Neurosurgery, University Hospitals Leuven, Leuven, Belgium; ^7^ Leuven Brain Institute (LBI), KU Leuven, Leuven, Belgium

**Keywords:** oxidative stress, GBM, bioinformatics, antioxidant phenotype, signatures, canonical GBM classification, transcription factors, prognosis

## Abstract

**Introduction:**

Aberrant reactive oxygen species (ROS) production is one of the hallmarks of cancer. During their growth and dissemination, cancer cells control redox signaling to support protumorigenic pathways. As a consequence, cancer cells become reliant on major antioxidant systems to maintain a balanced redox tone, while avoiding excessive oxidative stress and cell death. This concept appears especially relevant in the context of glioblastoma multiforme (GBM), the most aggressive form of brain tumor characterized by significant heterogeneity, which contributes to treatment resistance and tumor recurrence. From this viewpoint, this study aims to investigate whether gene regulatory networks can effectively capture the diverse redox states associated with the primary phenotypes of GBM.

**Methods:**

In this study, we utilized publicly available GBM datasets along with proprietary bulk sequencing data. Employing computational analysis and bioinformatics tools, we stratified GBM based on their antioxidant capacities and evaluated the distinctive functionalities and prognostic values of distinct transcriptional networks in silico.

**Results:**

We established three distinct transcriptional co-expression networks and signatures (termed clusters C1, C2, and C3) with distinct antioxidant potential in GBM cancer cells. Functional analysis of each cluster revealed that C1 exhibits strong antioxidant properties, C2 is marked with a discrepant inflammatory trait and C3 was identified as the cluster with the weakest antioxidant capacity. Intriguingly, C2 exhibited a strong correlation with the highly aggressive mesenchymal subtype of GBM. Furthermore, this cluster holds substantial prognostic importance: patients with higher gene set variation analysis (GSVA) scores of the C2 signature exhibited adverse outcomes in overall and progression-free survival.

**Conclusion:**

In summary, we provide a set of transcriptional signatures that unveil the antioxidant potential of GBM, offering a promising prognostic application and a guide for therapeutic strategies in GBM therapy.

## Introduction

Reactive oxygen species (ROS) are the by-products of multiple cellular and metabolic processes. It is widely acknowledged that low levels of ROS promote cell growth and differentiation (1), whereas higher levels of ROS can impart fatal damage to cellular components and trigger cell death (2). Cancer cells display basally high levels of ROS as compared to their normal counterparts. Several intrinsic genetic and metabolic alterations driving the malignant state, including oncogene expression and rewiring of major metabolic pathways, cause an imbalance in the cellular redox tone shifting the balance in favor of a pro-oxidant state, a condition known as “oxidative stress”. To withstand oxidative stress and avoid irreparable damage to vital entities, malignant cells increase their capacity to detoxify the excessive production of ROS. As a consequence, failure to maintain a functional cellular antioxidant defense system causes inevitably ROS-driven cellular damage that results in cell demise which can occur through different regulated cell death (RCD) modalities (3–7). Efforts to maintain redox homeostasis in cancer cells can be challenged by the local tumor microenvironment, upon invasion of malignant cells in the bloodstream, which is notoriously more oxidizing, or colonization to a secondary site (8). Emerging data indicate that non-genetic mechanisms that contribute to tumor cell heterogeneity and drug resistance, involve transcriptional reprogramming of antioxidant response networks, which endorse cancer cells with an increased ability to cope with intrinsic and extrinsic oxidative stress (9–11). However, given the double-edged function of ROS, it remains unclear which changes in the intracellular redox tone are associated with various stages of malignancy, and when and how they contribute to the maintenance of cancer cell’s plasticity.

This concept seems particularly applicable to glioblastoma multiforme (GBM), the most aggressive brain neoplasm hallmarked by high heterogeneity, which drives treatment resistance and tumor recurrence (12). The high metabolic rate of GBM leads to the generation of excessive amounts of ROS and metabolic adaptation in these cells plays an essential role in resistance to oxidative stress-induced cell death. Congruently, in response to chemo (temozolomide - TMZ) or radio-therapy GBM activates redox-sensitive transcription factors, including nuclear factor-κB (NF-κB), nuclear factor erythroid 2 p45-related factor 2 (NRF2), or HIF-1 that cooperate to support cancer cell survival and progression through cell-intrinsic and -extrinsic mechanisms (13, 14). Hence, how GBM strives to maintain redox pathways promoting tumorigenesis and resistance to anticancer therapies, while avoiding oxidative stress-induced killing remains an outstanding question.

In this perspective, it would be valuable to explore whether gene regulatory networks can capture different redox states are associated with the main GBM phenotypes. If so, this could help in understanding how glioma cell plasticity and redox signaling are co-regulated. Furthermore, given that several clinically available anticancer treatments kill cancer cells by directly or indirectly inducing lethal levels of ROS (14), an ‘a priori’ knowledge of the cancer cell’s antioxidant capacity based on the co-expression of redox-regulating genes, may provide an indicator of the propensity of a ROS-inducing drug/treatment to be effective and be therefore clinically informative. Identifying such a gene signature redox-based classifier could provide an additional tool to predict GBM patient responses to therapies. Here we perform an in silico analysis to define a redox-gene expression signature that could provide useful insights into the propensity of a particular GBM state to undergo lethal oxidative stress.

## Materials and methods

### Software, signature of interest, datasets and workflow

**Figure 1:**
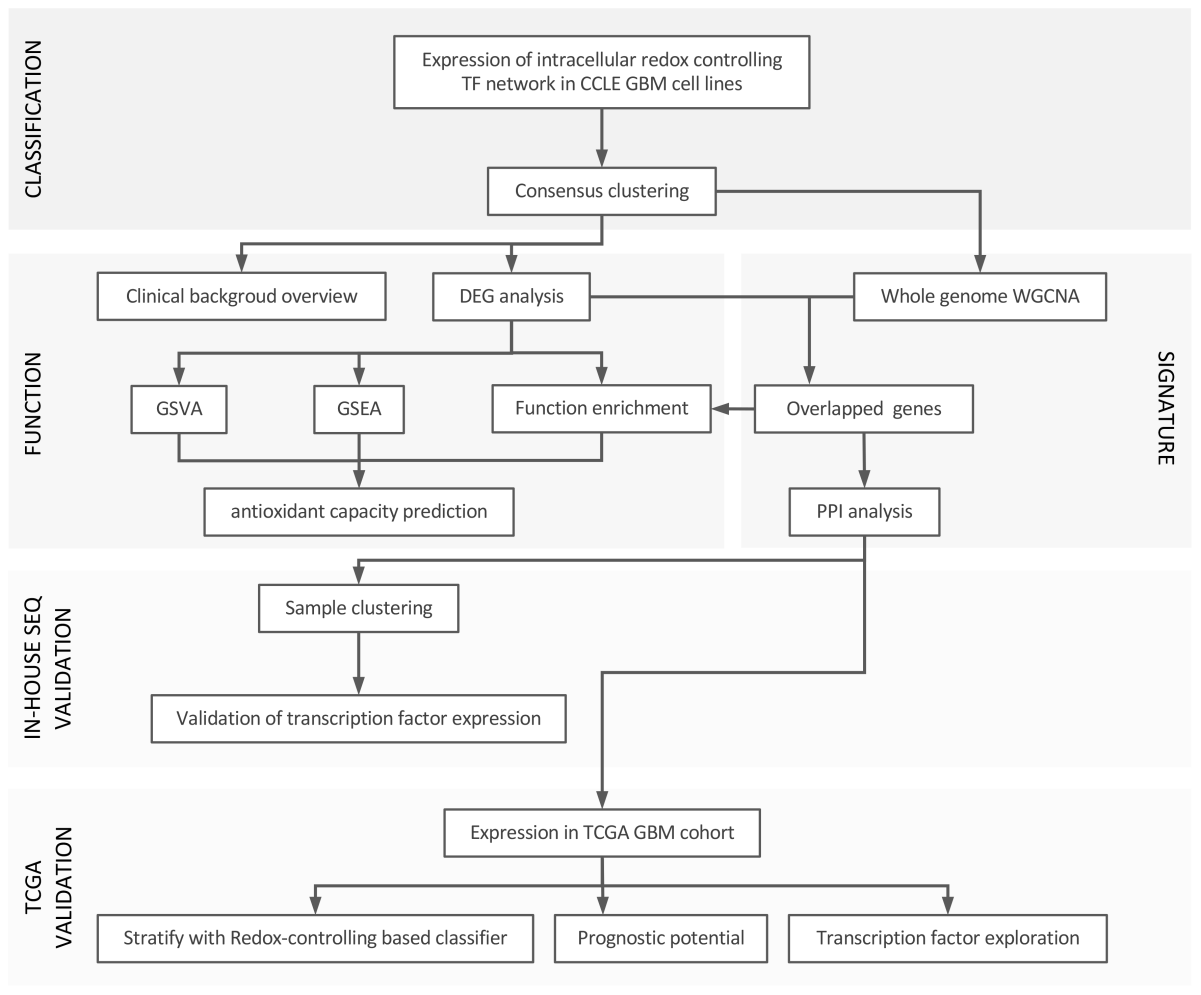
Workflow of the study. In general, we divided our analysis into three parts: 1) set up of the classification using redox homeostasis controlling transcription factor network; 2) function annotation and signature establishment; 3) validation by in-house RNA-seq and TCGA-GBM project, and prognosis analysis with TCGA GBM cohort.

Unless stated otherwise, R 4.2.2 and RStudio were used to perform our analysis based on the signature of redox controlling transcription factor network reported in a recent paper (15). Cancer cell line encyclopedia (CCLE) database, The Cancer Genome Atlas Program (TCGA) database and The Genotype-Tissue Expression (GTEx) portal were used. All the original data was transformed and parsed with the R package tidyverse. We used GBM cell lines (n = 59) from the CCLE database and downloaded raw data from weblink: https://sites.broadinstitute.org/ccle/. The background of each cell line was screened manually to confirm the pathological diagnosis of GBM. Related information was extracted to build the clinical profile of the cohort. We utilized the in-house RNA-seq data of patient-derived GBM cell lines, which were isolated and maintained primarily from clinical GBM samples (n = 41; generated under study number S59804 and S61081), and the GBM TCGA database (n = 168) as the validation cohorts for our newly defined classifier and performed prognosis analysis with the TCGA cohort. Expression of normal tissue from GTEx was used to compensate for the lack of normal tissue data in the TCGA project. The workflow of the study can be found in **Figure 1**.

### Consensus clustering and expression heatmap of the signature

**Figure 2:**
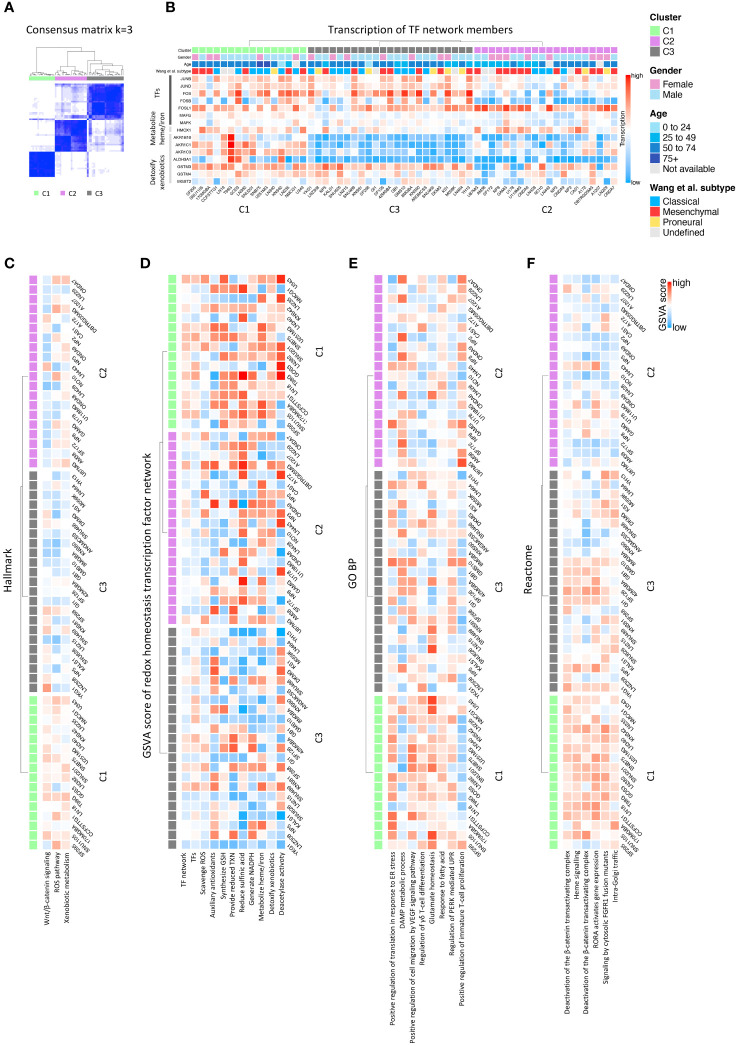
Consensus clustering of GBM CCLE cohort and function exploration. **(A)** Consensus matrix showing three distinct GBM clusters; **(B)** Major diversly expressed genes in the input signature; **(C)** Significant pathways evaluated by GSVA scoring in Hallmark database; **(D)** GSVA scores of each antioxidative pathways in the redox homeostasis transcription factor network; **(E)** Significant pathways evaluated by GSVA scoring in GO BP database; **(F)** Significant pathways evaluated by GSVA scoring in Reactome database.

We used CCLE GBM cohort for consensus clustering analysis. The expression of genes from the signature of redox controlling transcription factor network (15) was extracted as input matrix for clustering. With R package ConsensusClusterPlus, consensus matrix was built and stability assessment was performed to seek the optimal k value. We also confirmed the optimal k value with the function embedded in the package. In the end, k value of 3 was selected based on the stability of the clusters (**Figure 2A**; **Supplementary Figures 1B–J**). We, thereafter, found the three distinct groups of cell lines as the major clusters defined in our classification system. R package ComplexHeatmap was used to derive the heatmap for hierarchical clustering.

### Functional annotation

Gene set variation analysis (GSVA) was first used to gain the score of antioxidant pathways described in our input signature. The score was calculated with the R package GSVA. Then, all the databases in the R package msigdbr were extracted for GSVA scoring to validate the consistency of the results. The results from the database included Hallmark, Gene Ontology (GO), Reactome, WikiPathways, BioCarta, the Pathway Interaction Database (PID), and Cancer Module (CM), which showed significant readout, were reported here. We built the heatmap with above mentioned R package to assign the function to each cluster. Next, we performed differential expression gene (DEG) analysis with R package DESeq2 on counts data to find the up-regulated and down-regulated genes in each cluster. Log2 fold-change of 0.5 and adjusted p value, derived from Benjamin-Hochberg correction, of 0.05 were set as the thresholds. R package EnhanceVolcano was used to generate the Volcano plot presenting differentially expressed genes. Gene set enrichment analysis (GSEA) was performed to assign the functions to the clusters. We used the function in R package clusterProfiler to obtain the result for this step.

### Weighted gene co-expression network analysis and protein-protein interaction network construction

WGCNA was performed on the top 5000 expressed genes with R package WGCNA. This is to maintain the performance of the algorithm, in the meantime, to acquire the correlated modules with higher accuracy. Soft power was calculated, and we selected 5 as the optimal soft power to emphasize strong correlations and reduce the weaker (**Supplementary Figure 3D**). A signed network type was used to detect the co-expression gene modules. We took the three clusters as one set of traits, bringing along with clinical features and canonical GBM classification. A trait-module correlation was then produced. We gathered the genes from the, either positively or negatively, significantly correlated with each grouping as another bundle of gene lists to distinguish the three newly defined clusters. Next, we extracted the overlapped genes in the lists obtained from DEG analysis and the lists from WGCNA analysis to elucidate the signatures of the antioxidant GBM classification. STRING (https://string-db.org) was used as the tool for PPI analysis. To maximize the findings, we have utilized the default setting of active interaction sources from the webtool. These sources of interactions include textmining, experiments, databases, co-expression, neighborhood, gene fusion and co−occurrence. Results were imported into Cytoscape for network presentation. Plug-ins, cytoHubba and ClueGO of Cytoscape were used to search for the hub genes and annotate the functions.

### Survival analysis

Overall survival (OS) and progression-free interval (PFI) data of GBM samples from the TCGA cohort was used as input for Kaplan-Meier survival analysis with R package survival and survminer. Categorically, we compared the samples with the different clusters C1-C3. Quantitatively, we calculated the GSVA scores of each signature in the whole cohort, and compared the prognostic values between the high and the low scores. For the single gene analysis, transcription factors in each signature were spotted by the transcription factor database: http://humantfs.ccbr.utoronto.ca/index.php and http://bioinfo.life.hust.edu.cn/AnimalTFDB4/#/. Expression status was illustrated by violin plots derived from R package ggplot2. Again, related R packages were used to examine the prognostic values. Finally, the hazard ratio was evaluated with the function from the same R packages.

## Results and discussion

### Establishment of a GBM classification based on distinct antioxidant gene network phenotypes

The canonical GBM classification system based on transcriptomic differences fails to provide the fundamental biological characteristics that can guide the therapeutic propensity of the cellular states. Recently, in a study using an in silico pathway-based classifier, GBM was clustered into four main biological subtypes, characterized by divergent metabolic states (e.g. mitochondrial, glycolytic, lipid) and neurodevelopmental axis (16). Interestingly, the mitochondrial GBM phenotype, relying on oxidative phosphorylation and associated with higher levels of intracellular ROS, exhibited higher responses to radiation, a clinically relevant, ROS-inducing therapy in GBM. These studies further portrayed that GBM metabolic heterogeneity, possibly linked to a differential redox-tone, is linked to clinical outcomes.

To define a classification system for GBM, based on the intrinsic ability of cells to detoxify ROS and maintain redox homeostasis, we initially explored the RNAseq dataset from the CCLE database. We performed consensus clustering using the signature consisting of genes regulated by members of the antioxidant transcription factor network (15) (see Materials and Methods, and **Supplementary Figure 1A**). GBM cell lines could be segregated into three main clusters labeled C1, C2, and C3 (**Figure 2A**). Significant definers of each cluster included members of the activator protein-1 (AP-1) family of transcription factors, and genes involved in heme or iron metabolism and the detoxification of xenobiotics (15). Analysis of the expression of these genes across the GBM cell lines showed they were expressed prevalently in the C1 cluster, whereas their expression was low to very low in the C2 and C3 clusters, respectively (**Figure 2A**; **Supplementary Figure 1A**). This suggests that the C1 GBM cluster express a transcriptional network endowed with more robust antioxidant ability, compared with C2 and C3 (**Figure 2B**). Hierarchical clustering analysis resulted in a similar segregation of cell lines (**Supplementary Figure 1**). The clinical background of the individual sample can be visualized in **Figure 2B**.

To gain further insight into the molecular signature of each cluster, we performed GSVA utilizing different databases. We started the analysis using the Hallmark 50 database, and identified the term ROS pathway together with the terms Wnt/β-catenin signaling, and xenobiotic metabolism differentiating the three clusters, thus validating the signature (**Figure 2C**). Further GSVA analysis using literature-driven annotation of genes (genes used in the signature) revealed that all the terms related to antioxidant functions (e.g. scavenge ROS, provide reduced thioredoxin (TXN), synthesis of glutathione (GSH), generate NADPH, metabolize heme/iron and detoxify xenobiotics) and transcription factors (TF) regulating and antioxidant response were highly enriched in C1 followed by C2 while poorly coexpressed in C3 (**Figure 2D**). To gain further insights into the molecular pathways potentially contributing to the difference in redox signature across the three clusters, we performed GSVA using gene ontology (GO) and pathway analysis. The GO analysis identified terms, such as the regulation of PERK-mediated UPR, glutamate homeostasis, and response to fatty acids, enriched in C1 (**Figure 2E**; **Supplementary Figures 2A, B**). Pathway analysis using different databases (Reactome, WikiPathways, BioCarta, PID, and CM) identified PERK, NRF2, ferroptosis, iron homeostasis, and cytokine pathways driving inflammation, as dominant pathways differentiating the three clusters (**Figure 2F**; **Supplementary Figures 2C–F**).

The co-existence of the PERK branch of UPR and NRF2 in C1 is congruent with the relevant role of this ER stress sensor in the resistance to oxidative stress in cancer cells (17). In line, PERK mediates the phosphorylation of NRF2 on Thr-80, which unleashes NRF2 from its inhibitory association with KEAP1 thereby favoring NRF2 nuclear translocation and boosting the transcription of the anti-oxidant response genes (18). These genes include heme-oxygenase-1 (HO-1), which generates the antioxidant bilirubin and glutamate-cysteine ligase-catalytic subunit (GCLC), which is essential for the synthesis of the major intracellular anti-oxidant glutathione (GSH) (18). Furthermore, the PERK-eIF2α-ATF4 axis of the UPR also contributes to the mitigation of oxidative stress in cancer cells by the ATF4-mediated increase in amino acid transport and metabolism (19–21). Among other targets of this pathway, the expression of the glutamate transporter SLC7A11, which exchanges glutamate for the import of cystine, increases the intracellular concentration of GSH. In line with this, multiple studies have indicated that attenuation of glutamate homeostasis leads to the accumulation of ROS (22). In conjunction, the terms glutamate homeostasis, iron/heme homeostasis, and ferroptosis are also enriched in C1. In line with this, recent studies linked the PERK-NRF2-HO-1 axis of the ER stress pathway to the modulation of ferroptosis (23). Additionally, the increased presence of terms related to NADP activity within the molecular function (MF) ontology and the pentose phosphate pathway (PPP) in C1 may also be correlated with the increased activation of NRF2 (15). Several NADP-related terms were highly enriched in C1 followed by C2 at the level of MF in the GO analysis (**Supplementary Figure 2A**). Active NRF2 is associated with increased glucose uptake, which is preferentially metabolized through PPP resulting in increased reducing equivalent capacity, via the production of NADPH (24). NADPH is required for and consumed during fatty acid synthesis and the scavenging of ROS.

Of note, despite exhibiting a dominant antioxidant transcriptional network, the GBM C1 cluster also showed an enrichment of several terms related to the production of protumorigenic/angiogenic cytokines, such as IL-6, IL-7, IL-9 (25) and vascular endothelial growth factor (VEGF) (26) (**Supplementary Figure 2D**). This could be linked to a chronic activation of the UPR, coupling the upregulation of the PERK-NRF2 antioxidant response pathway, with the stimulation of NF-κB mediated proinflammatory cytokines (27, 28), an interesting conjecture to be explored in future functional studies. In GBM, β-catenin and components of the Wnt pathway are commonly found to be overexpressed, contributing to cancer initiation, proliferation and invasion (29). It’s worth noting that ROS, acting as signaling molecules, also exert control over the Wnt–β-catenin signaling pathway (30). Together, this suggests that the GBM C1 cluster deploys the ability to detoxify potentially harmful ROS while maintaining a redox-tone supporting protumorigenic cell intrinsic and extrinsic signaling pathways.

Taken together, this analysis portrays that compared with the other two clusters, the C1 identifies a GBM entity hallmarked by a heightened antioxidant and protumorigenic potential.

### Identification of transcriptional networks governing differentiated antioxidant potentials

**Figure 3:**
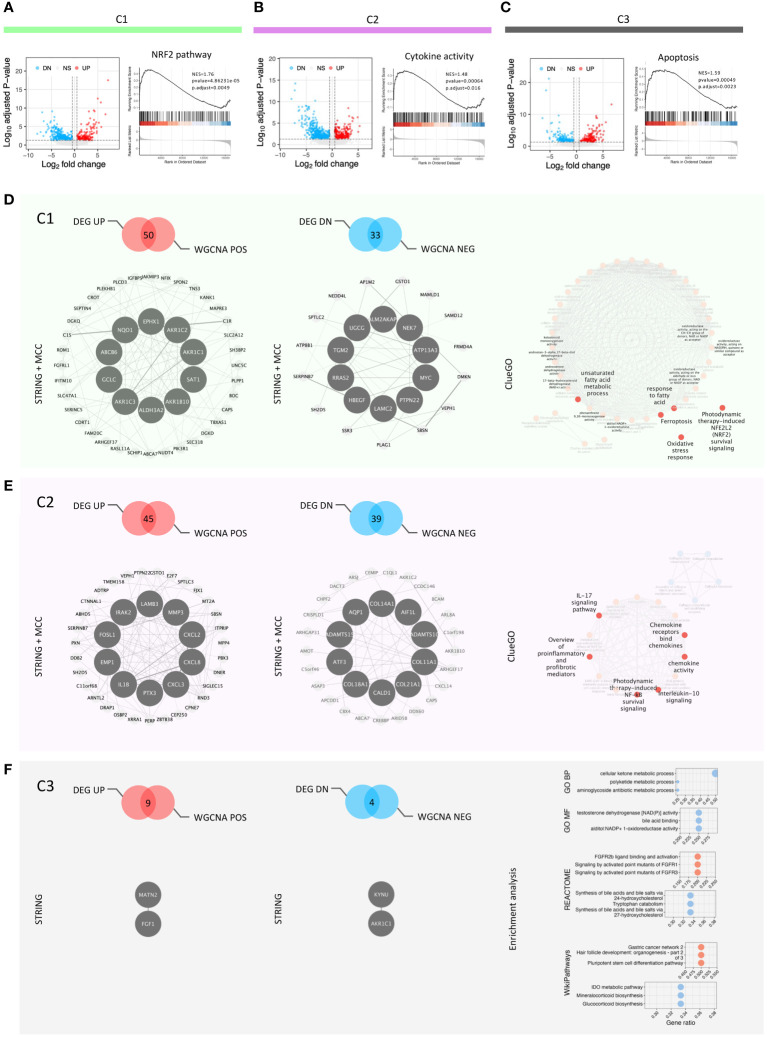
DEG analysis, GSEA and redox-based signatures. **(A–C)** Volcano plots showing DEG among the clusters and the key GSEA results in each cluster; **(D)** Feature panel of C1: positive and negative signatures, hub genes and function enriched; **(E)** Feature panel of C2: positive and negative signatures, hub genes and function enriched; **(F)** Feature panel of C3: positive and negative signatures, hub genes and function enriched.

With the aim of identifying transcriptional networks with hub genes regulating the signature of each cluster, we integrated DEG analysis with the WGCNA method. DEG analysis identified 173, 356 and 220 genes upregulated, while 410, 684 and 174 genes downregulated in C1, C2 and C3, respectively (**Figures 3A–C**; **Supplementary Table 1**). The results of GSEA on the upregulated gene set across each cluster were in line with GSVA, identifying genes involved in the NRF2 pathway, fatty acid metabolism and suppressors of ferroptosis (**Supplementary Figure 3B**) as highly expressed in C1 (**Figure 3A**; **Supplementary Figure 3A**). In contrast, C2 clustered genes of several inflammatory pathways such as response to LPS, cytokine active and IFN-α/γ response (**Figure 3B**; **Supplementary 3C**). Interestingly C3, which exhibits a limited ability to scavenge ROS, showed an enrichment for apoptosis pathway (**Figure 3C**).

Next, we used WGCNA to build gene modules of significantly correlating genes (**Supplementary Figures 3E, F**), and then evaluated how these modules relate to the clustering pattern by calculating Pearson correlations between each module and cluster (**Supplementary Figure 3G**, **Supplementary Table 2**). We integrated the genes from modules correlating closely with each cluster and their DEGs (**Figures 3D–F**). The integrated gene lists were used as the input for STRING analysis to gain the protein-protein interaction network (PPI network) of each cluster. The PPI networks were incorporated into the Cytoscape software to characterize the hub genes regulating this network. The top ten hub genes were identified for each cluster, except C3, using the cytoHubba plug-in in Cytoscape (**Figures 3D, E**). The low number of input genes for C3 hindered this analysis (**Figure 3F**). We defined the coexisting genes in both positively correlated genes from WGCNA and up-regulated genes from DEG analysis as the signatures of the three clusters (**Supplementary Table 3**). Further, to identify the transcription factors in each signature, we utilized the database as described in the method section (**Supplementary Figure 3H**). We identified one [*NFIX* (31, 32)], six [*ZBTB38* (33), *ARNTL2* (34), *E2F7* (35), *PBX3* (36), *FOSL1* (37, 38), and *DRAP1*), and one (*LHX9* (39)] transcription factors in cluster C1, C2 and C3, respectively, potentially regulating the cluster (**Supplementary Figure 3I**). All these transcription factors are known to have a role in the development and progression of GBM. Moreover, they are either linked directly or indirectly in regulating ROS-mediated signaling pathways. To characterize the functionality of these integrated genesets, we used ClueGo which incorporates different databases to identify pathways these genes are enriched in (**Figures 3D, E**). The results of ClueGo were in line with GVSA and GSEA analysis, further validating our observation that C1 has the hallmark of an antioxidant phenotype, C2 is associated with an inflammatory phenotype, while C3 is characterized by a propensity to undergo ROS-mediated apoptosis.

We then tested whether the derived C1, C2 and C3 signatures correlated with the canonical classification of classical, mesenchymal and proneural GBM (40) (**Supplementary Figure 4**). Of note, the classical subtype was distributed mainly across C1, C2 and C3, suggesting that classical GBM are heterogenous in their redox homeostasis, likely depending on factors, such as the stage of the disease, mutational status, etc. Remarkably, the mesenchymal GBM subtype showed a major distribution in C2 (**Supplementary Figure 4A**). This observation was further validated statistically using a GSVA-based model of correlation (**Supplementary Figure 4B**). It has been previously shown that mesenchymal cells are associated with a high ROS index, which can lead to chronic inflammation eventually promoting cell growth (41). The proneural subtypes showed the highest distribution in C3 suggesting that proneural cell types have the weakest ROS-defending potential and could be targeted by ROS-inducing therapies. However the proneural phenotype is known to switch to the mesenchymal phenotype as an adaptive response in the presence of excess ROS (41, 42).

Next, we validated our signature with an in-house bulk RNAseq dataset derived from GBM patient-derived cell lines (PDCLs). Using the derived antioxidant network signatures, we could cluster these PDCLs into three distinct groups (**Supplementary Figure 3J**). Of note, analyzing the expression of the transcription factors associated with clusters C1, C2, and C3, we could demonstrate similar trends, with an expression of *ZBTB38*, *ARNTL2*, *E2F7*, *FOSL1*, and *DRAP1* high in C2 and *LHX9* high in C3 (**Supplementary Figure 3K**). Moreover, analyzing the correlation of C1, C2 and C3 signatures with the canonical subtype showed similar trend with PDCLs in mesenchymal subtypes showing major distribution in C2 (**Supplementary Figures 4C, D**).

Hence, the transcription factors identified for each gene network across different clusters may be predictive of GBM types that can benefit from particular ROS-induced therapeutic approaches. However, these observations require thorough functional investigations both *in vitro* and *in vivo* settings to confirm this assumption. The intriguing correlation between C2 with mesenchymal suggests that genomic-based classification of GBM can be associated with a differential redox homeostasis and antioxidant potential. This connection can be harnessed for targeted therapeutic approaches.

### Prognostic significance of the redox-based classifier and the transcription factors associated

**Figure 4:**
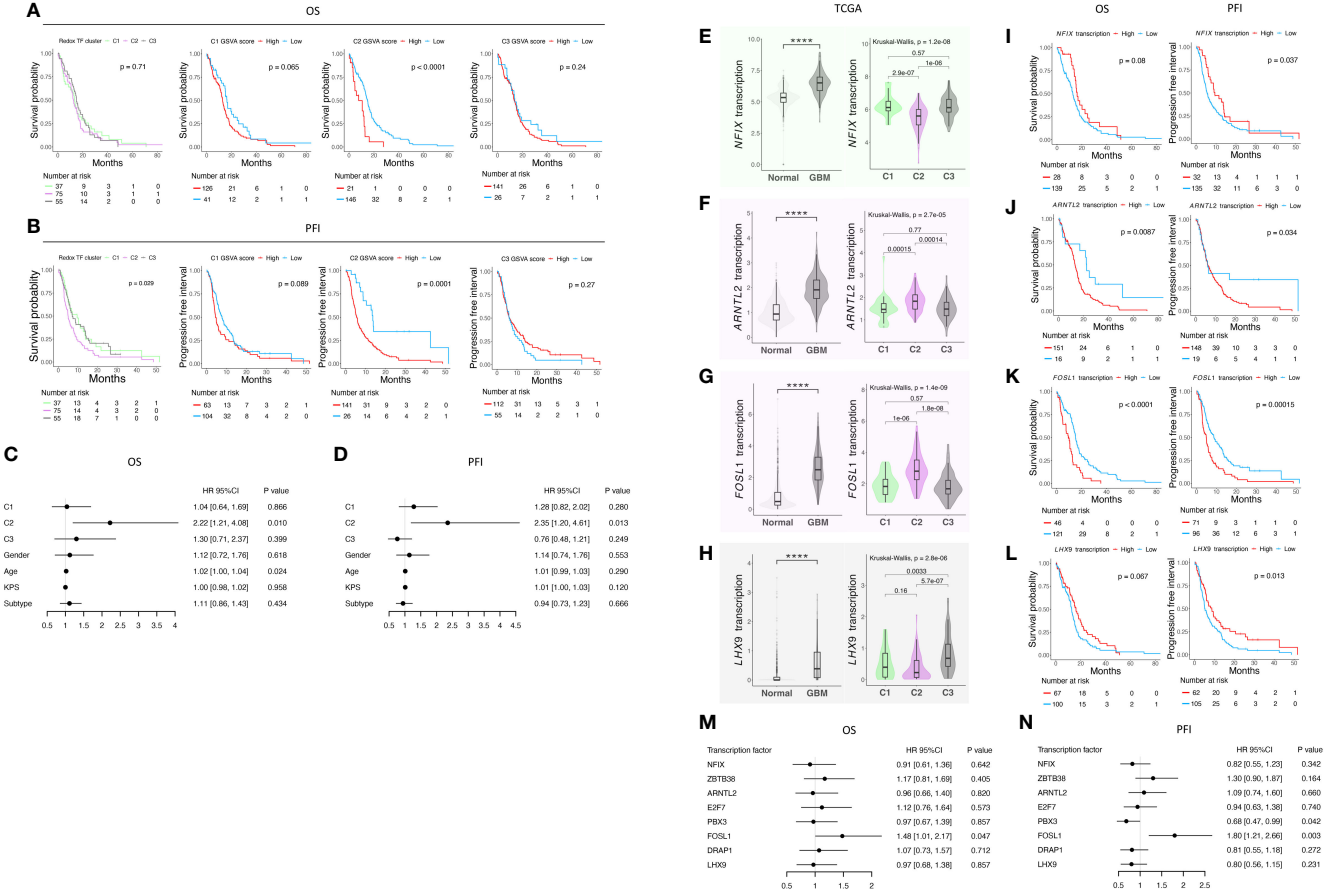
Survival analysis of the classification in TCGA GBM cohort and analysis of transcription factor in the signature of each cluster. **(A)** K-M curves of OS; **(B)** K-M curves of PFI; **(C)** Table of hazard ratio on OS; **(D)** Table of hazard ratio on PFI; **(E)** *NFIX* expression status and **(F)** its prognostic value; **(G)** *ARNTL2* expression status and **(H)** its prognostic value; **(I)** *FOSL1* expression status and **(J)** its prognostic value; **(K)** *LHX9* expression status and **(L)** its prognostic value; **(M)** Table of hazard ratio on OS; **(N)** Table of hazard ratio on PFI. **** indicates a p-value < 0.0001.

We utilized the TCGA-GBM dataset to examine whether our antioxidant transcription factor network-based classification system has any prognostic value. First, based on the signatures, GSVA scores of C1-C3 were calculated for each sample of TCGA-GBM study. Using these scores, we could divide the patients into three different sub-cohorts (**Supplementary Figure 5A**). Next, we characterized the prognostic value of each cluster both at the levels of overall survival (OS) (**Figures 4A, C**) and progression-free interval (PFI) (**Figures 4B, D**). Interestingly, C2 showed significantly worse prognosis both at the level of OS and PFI in both categorical and quantitative fashions (**Figures 4A, B**). According to the multivariate analysis, C2 showed a comparatively worse OS in GBM patients with a hazard ratio (HR) of 2.22 and a p-value of 0.010 (**Figure 4C**). Also, at the level of the PFI, C2 under-performed the other two phenotypes with an HR of 2.35 and a p-value of 0.013 based on multivariate survival analysis (**Figure 4D**).

We then focused on the transcription factors in the signature and wondered if they would provide more insights regarding GBM prognosis. We analyzed their expression in the TCGA-GBM cohort (**Figures 4E–H**; **Supplementary Figure 5B–E**). We found that all the transcription factors herein are differentially expressed compared with normal tissue. Except for ZBTB38 (**Supplementary Figure 5B**), all the other transcription factors are up-regulated in GBM, pointing to the re-design of redox-related mechanisms either due to the intrinsic ability of cancer cells or driven by the tumor microenvironment. We then checked the expression of each gene among the three clusters, and their prognostic value at the single gene level was underpinned (**Figures 4I-N**; **Supplementary Figures 5B–E**). We found that *FOSL1* is a potent predictor of both OS and PFI prognosis (**Figure 4K**). Lower expression of *FOSL1* indicates a better prognosis in GBM patients. Our finding shows that the patients stratifying in the C2 phenotype may have a preferable OS and PFI, and *FOLS1* can serve as a single gene biomarker predicting the survival of GBM patients.

**Figure 5:**
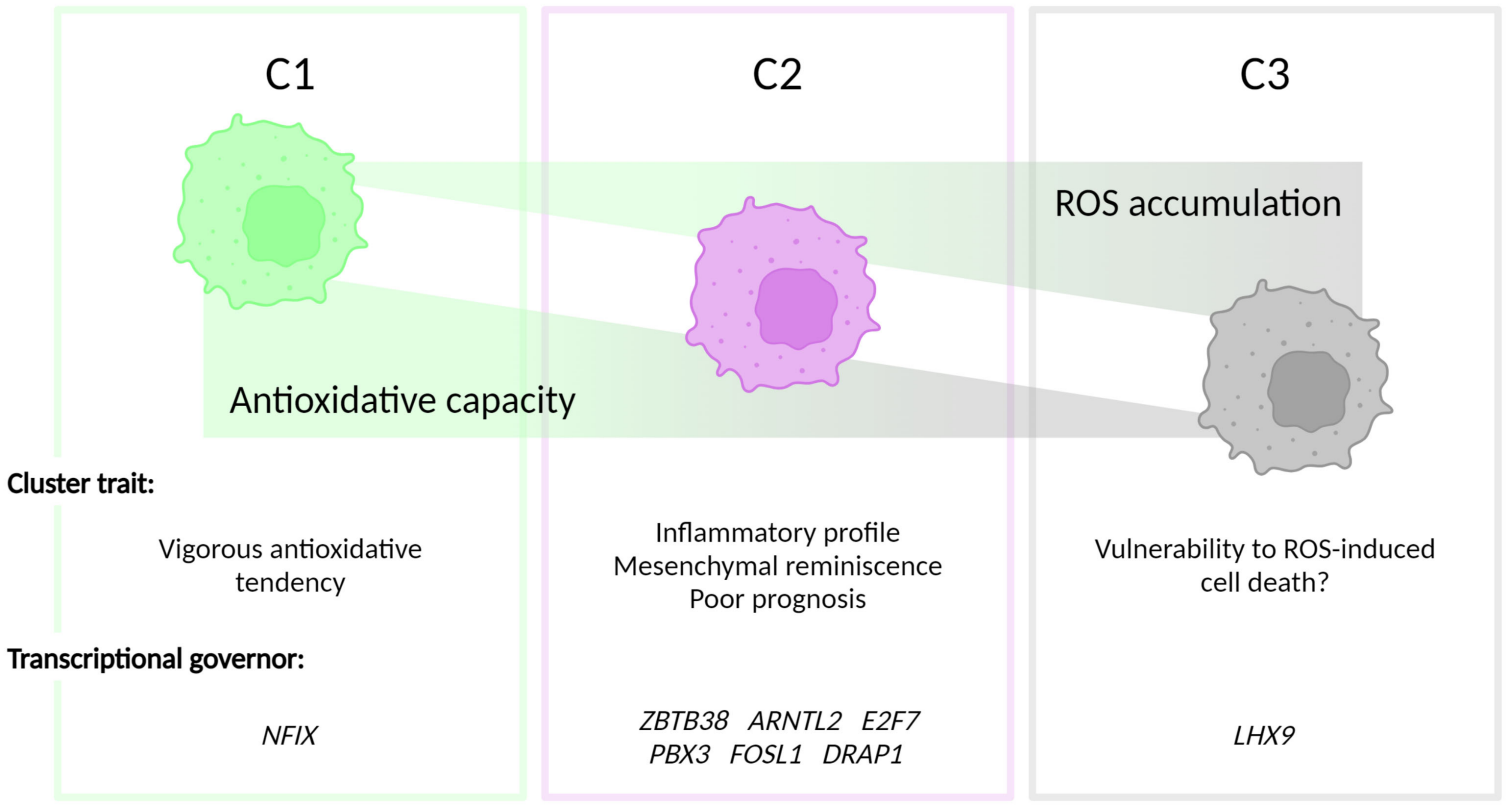
Figurative summary. GBM can be clustered into three groups, termed C1, C2 and C3, depending on the intrinsic antioxidative capacities. C1 is characterized by the strongest antioxidative potential. C2 has be discovered with the inflammatory phenotype and a correlation with mesenchymal GBM subtype. The antioxidation is dampened in C3, which, hypothetically, contributes to the vulnerability to ROS-triggered cell death in this cluster.

In summary, we report the prognostic value of the classification of the redox homeostasis controlling network (**Figure 5**). Interestingly, C2 defined by the new classifier is adequate to predict poor OS. Moreover, patients, who are classified as C2 phenotype, showed worsened PFI. Considering the function discovered in the enrichment analysis, C1 is marked as a cluster with a higher antioxidant potential compared with C2 and C3. In C2, a moderate level of ROS may function as a signaling molecule promoting GBM progression. The C3 phenotype has the weakest antioxidant potential and GBM clustered in this group may succumb to ROS-induced cell death. Among the transcription factors in the C2 signature, *FOSL1* demonstrates a prognostic value in both OS and PFI. This suggests that *FOSL1* under-expression is an independent factor linked to longer OS and delayed tumor progression. Interestingly, *FOSL1* has been shown to boost the transition of proneural-to-mesenchymal via NF-κB signaling (37). In pancreatic cancer, metastasis can be overcome by the suppression of FOSL1 expression by SMAD4 (43). Of note, *FOSL1* expression has been functionally related to cancer angiogenesis and vascularization, suggesting that reprogramming of the redox tone in this GBM cluster may be associated with its heightened neovascularization potential.

In conclusion, our analysis shows that the C2 cluster is closely correlated with mesenchymal GBM. Due to the pervasive angiogenic phenotype of the mesenchymal subtype of GBM, *FOSL1* could be an interesting target for future studies assessing the biological and therapeutic function of this transcription factor in GBM.

## Data availability statement

The original contributions presented in the study are included in the article/**Supplementary Material**. Further inquiries can be directed to the corresponding authors.

## Author contributions

YY: Writing – review & editing, Writing – original draft, Visualization, Validation, Investigation, Formal analysis, Data curation. SM: Writing – review & editing, Writing – original draft, Validation, Formal analysis. FS: Writing – review & editing, Resources, Funding acquisition. SV: Writing – review & editing, Supervision, Funding acquisition. PA: Writing – review & editing, Writing – original draft, Supervision, Funding acquisition, Conceptualization.

## Supplementary material

Supplementary Figure 1
**(A)** Multiple clustering algorithms underscore the robustness of antioxidative network-based classification. Expression heatmap and hierarchical clustering of antioxidative signature; **(B–J)** Consensus clustering with multiple k values.

Supplementary Figure 2 Heatmap of GSVA scoring biological function database. **(A)** GSVA score heatmap of GO MF database; **(B)** GSVA score heatmap of GO CC database; **(C)** GSVA score heatmap of WikiPathways database; **(D)** GSVA score heatmap of BioCarta database; **(E)** GSVA score heatmap of PID database; **(F)** GSVA score heatmap of CM database.

Supplementary Figure 3
**(A)** GSEA of C1; **(B)** Overlapping of ferroptosis signature with C1 signature; **(C)** GSEA of C2; **(D)** Soft power selection in WGCNA; **(E, F)** WGCNA modules presentation; **(G)** Module-trait correlations; **(H)** Transcription factors in each cluster signature and **(I)** their expression status in CCLE cohort; **(J)** redox clusters assignment of in-house GBM RNA-seq; **(K)** Expression of featured transcription factors in in-house cohort. SIG: signature.

Supplementary Figure 4 Bridging antioxidative clusters with GBM canonical classification. **(A)** Alluvial plot showing the cluster-subtype connection in CCLE cohort; **(B)** Correlation analysis of GSVA scores of signatures of antioxidative cluster and GBM canonical subtypes in CCLE cohort. **(C)** Alluvial plot showing the cluster-subtype connection in in-house cohort; **(D)** Correlation analysis of GSVA scores of signatures of redox cluster and GBM canonical subtypes in in-house cohort.

Supplementary Figure 4
**(A)** Subtype assignment in GBM samples from TCGA project; **(B)** *ZBTB38* expression status and its prognostic value; **(C)** *PBX3* expression status and its prognostic value; **(D)** *E2F7* expression status and its prognostic value; (E) *DRAP1* expression status and its prognostic value.

Supplementary Figure 5
**(A)** Subtype assignment in GBM samples from TCGA project; **(B)** *ZBTB38* expression status and its prognostic value; **(C)** *PBX3* expression status and its prognostic value; **(D)** *E2F7* expression status and its prognostic value; (E) *DRAP1* expression status and its prognostic value.
