# Supplementary material for: Antioxidant network-based signatures cluster glioblastoma into distinct redox-resistant phenotypes
Source: Front Immunol. 2024 Apr 18;15:1342977. doi: 10.3389/fimmu.2024.1342977 (PMC11063242; doi:10.3389/fimmu.2024.1342977)

Supplementary Figure 1

(A)

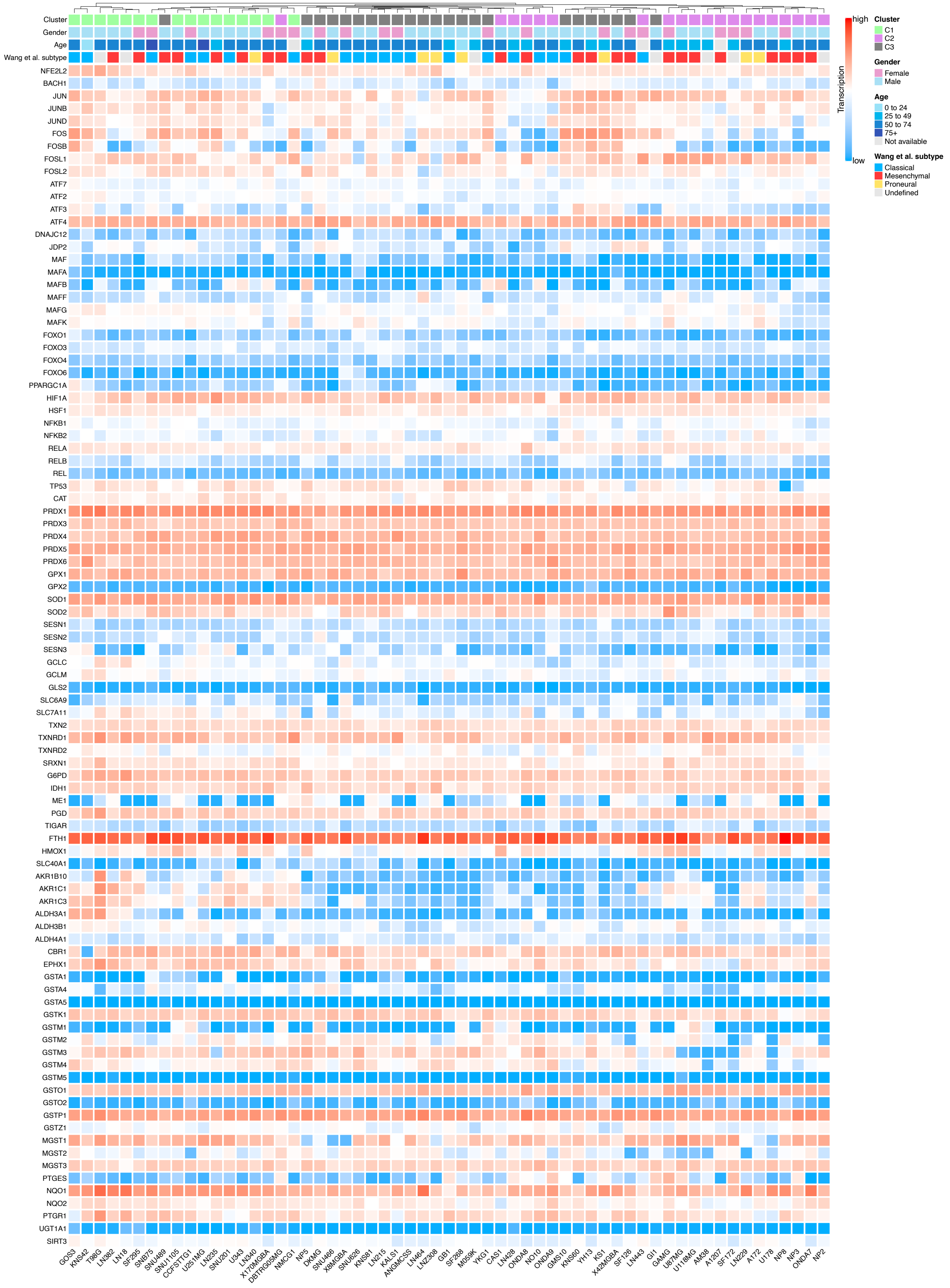

Supplementary Figure 1 (continued)

(B)

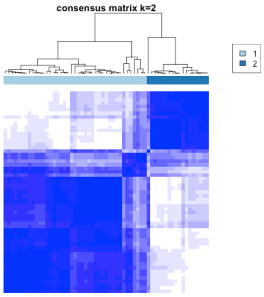

(C)

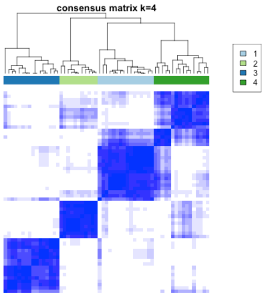

(D)

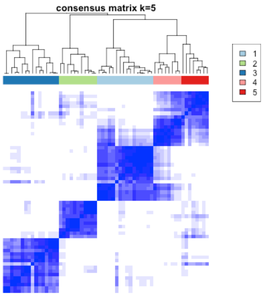

(E)

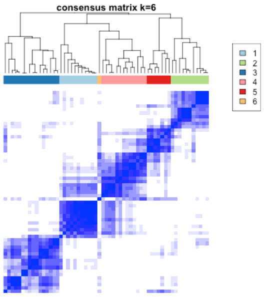

(F)

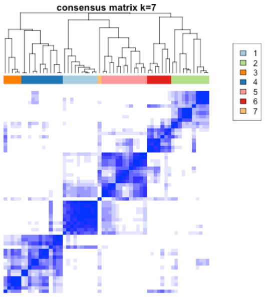

(G)

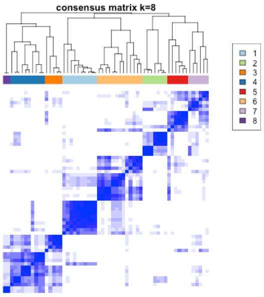

(H)

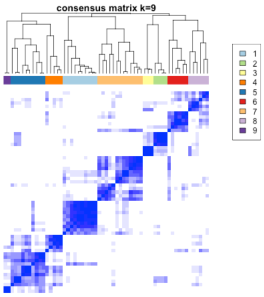

(I)

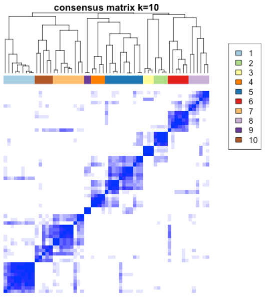

(J)

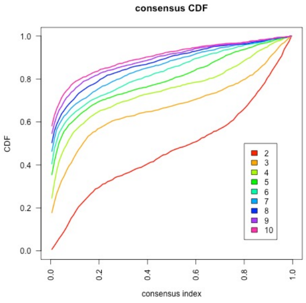

Supplementary Figure 2

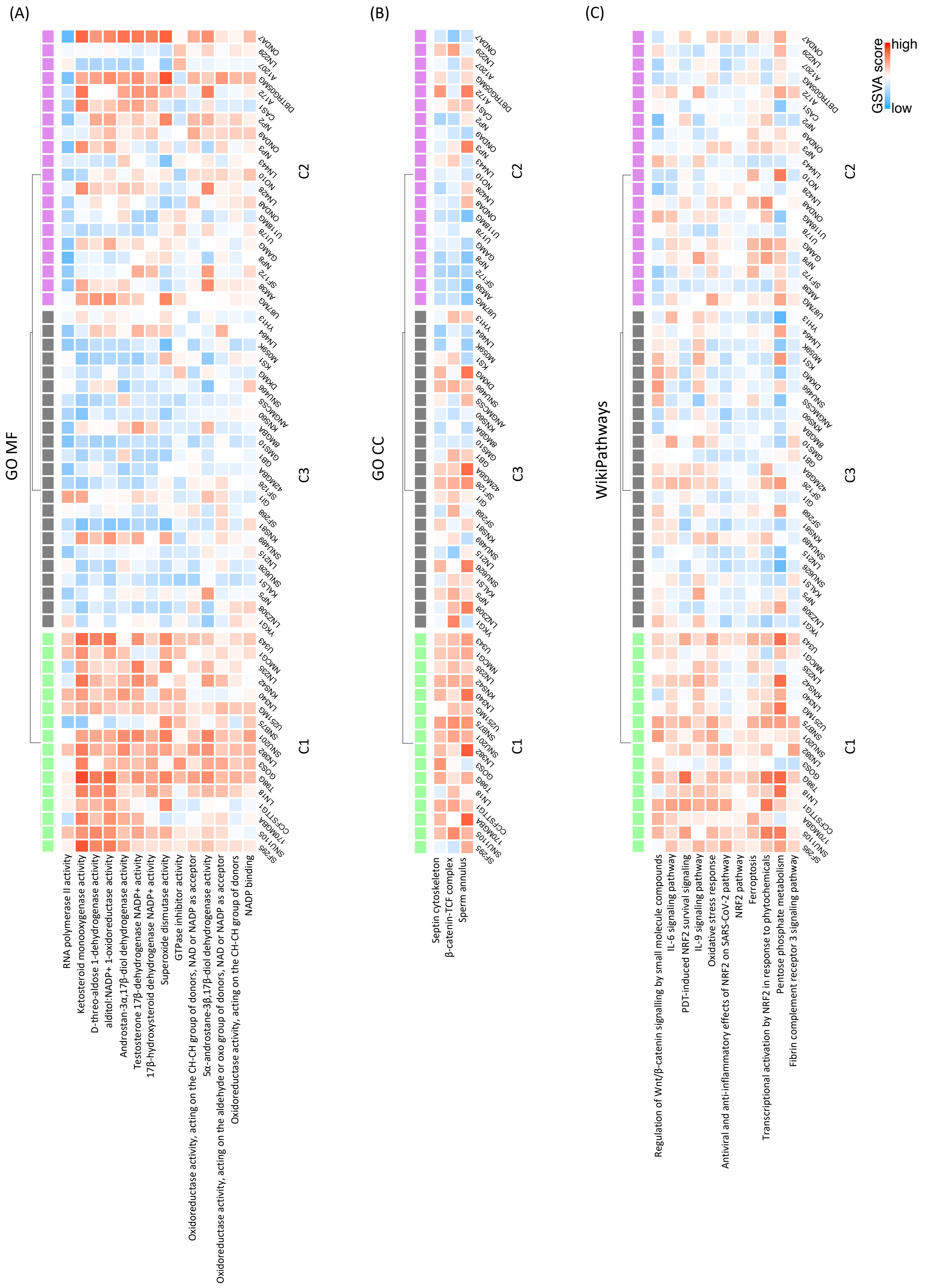

Supplementary Figure 2 (continued)

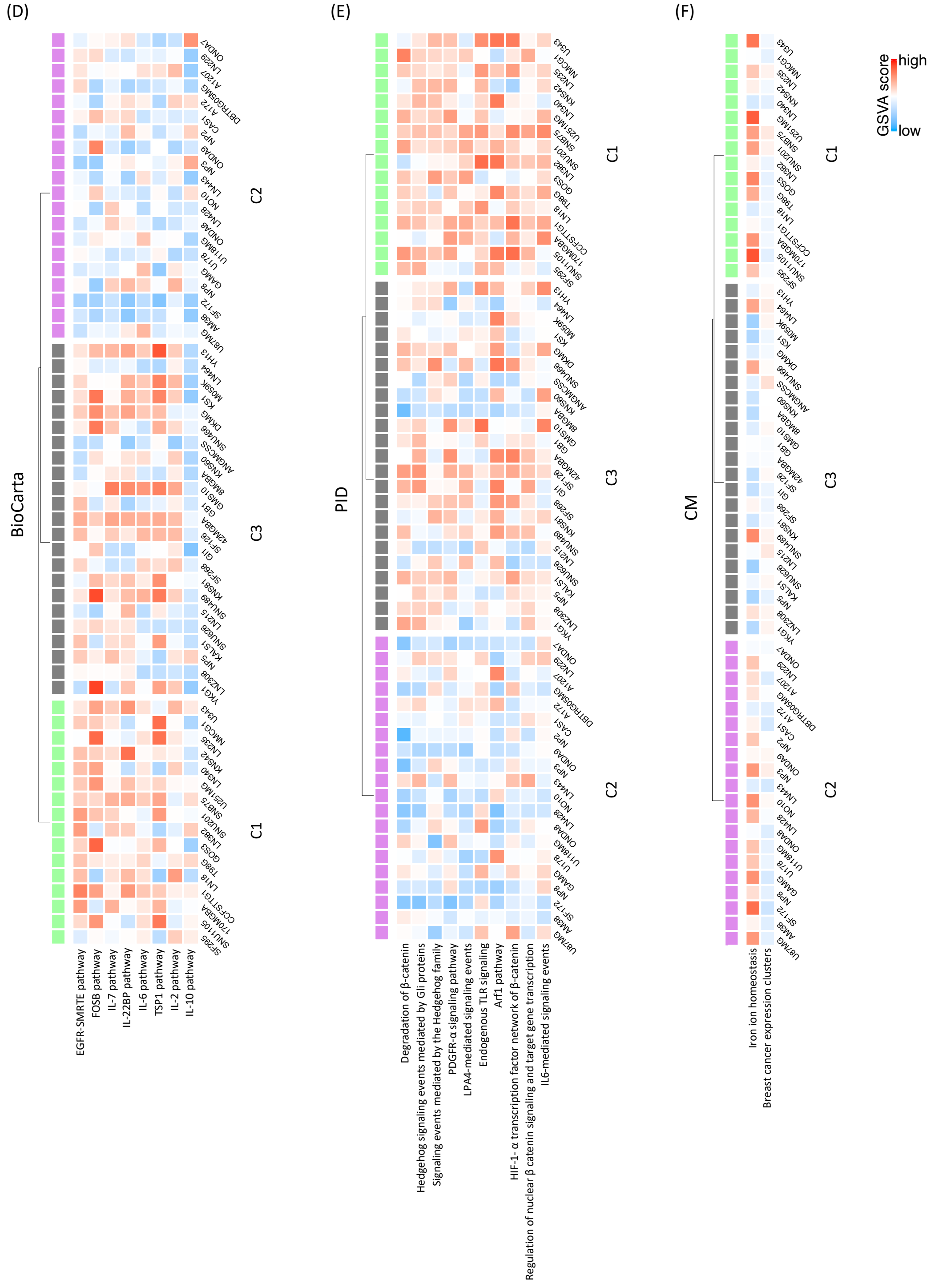

Supplementary Figure 3

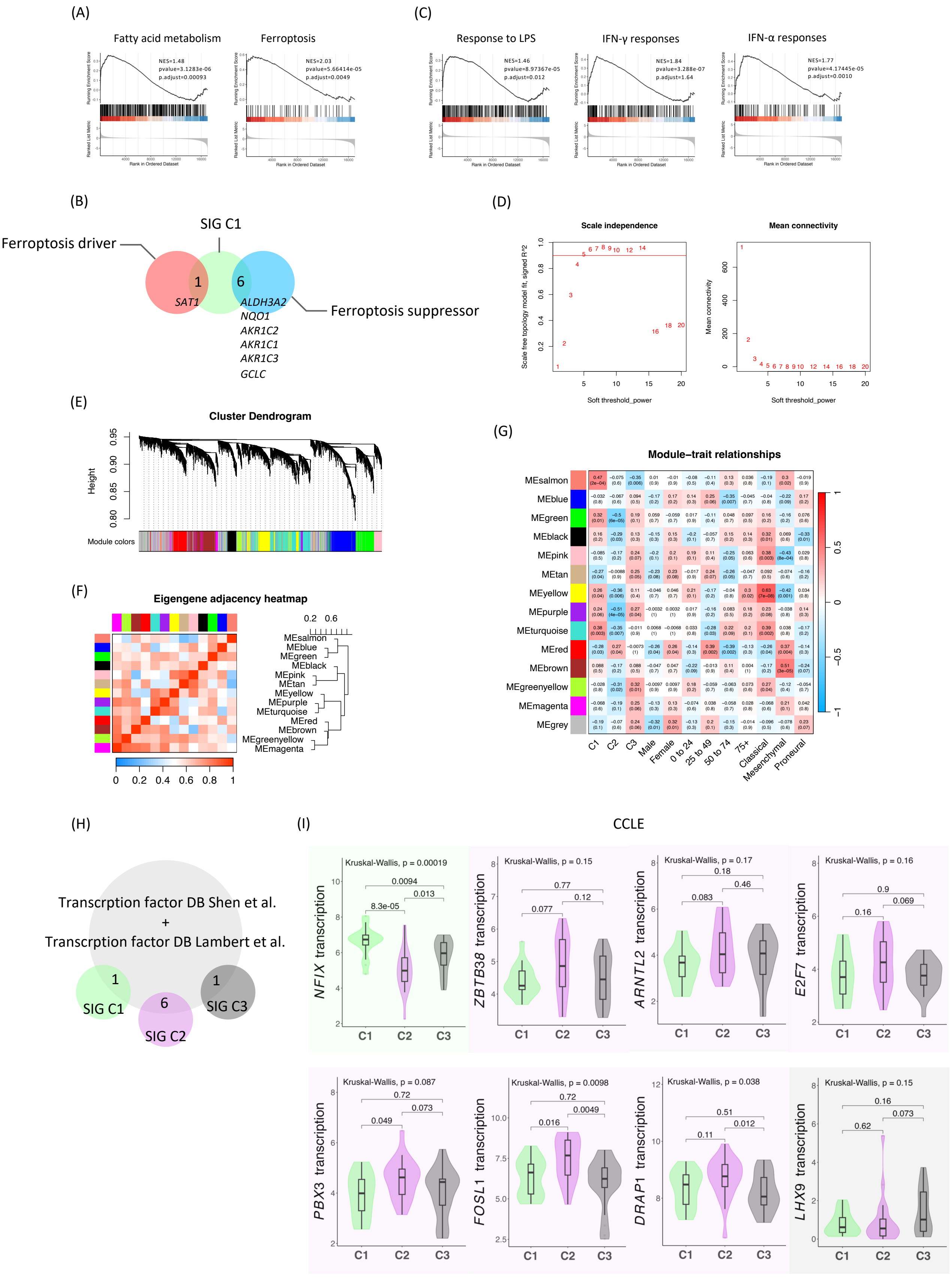

Supplementary Figure 3 (continued)

(J)

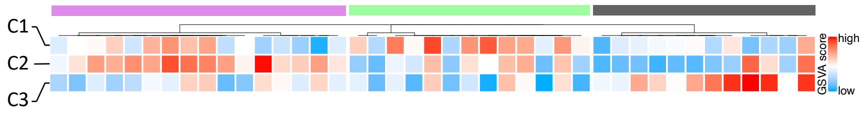

(K)

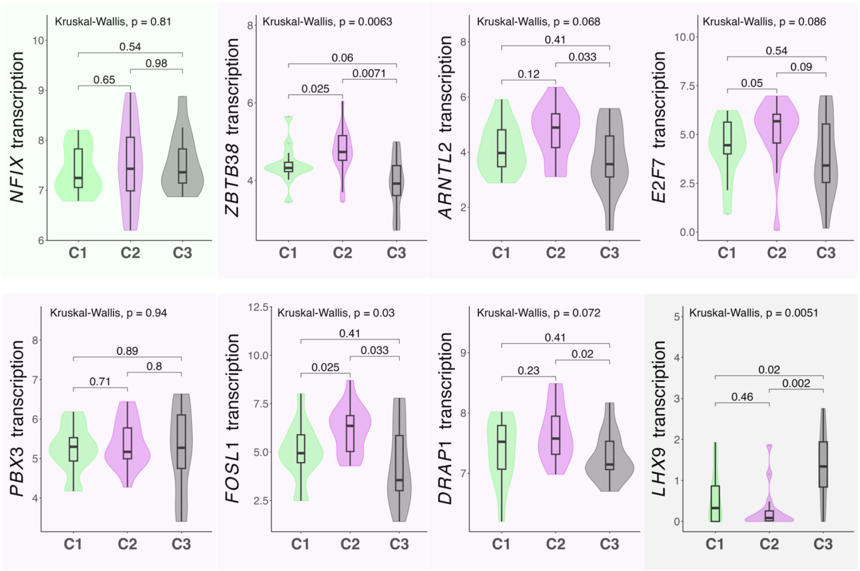

Supplementary Figure 4

CCLE

(A)

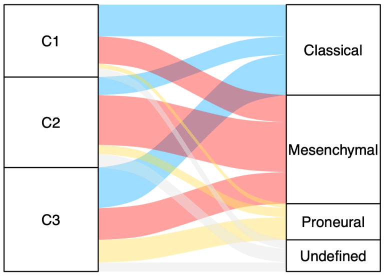

(B)

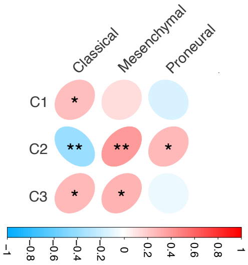

In-house

(C)

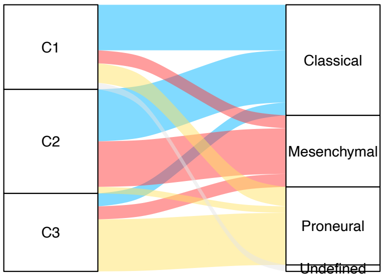

(D)

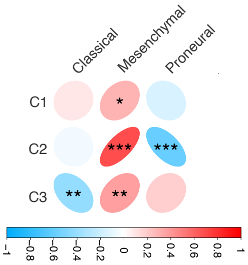

Supplementary Figure 5

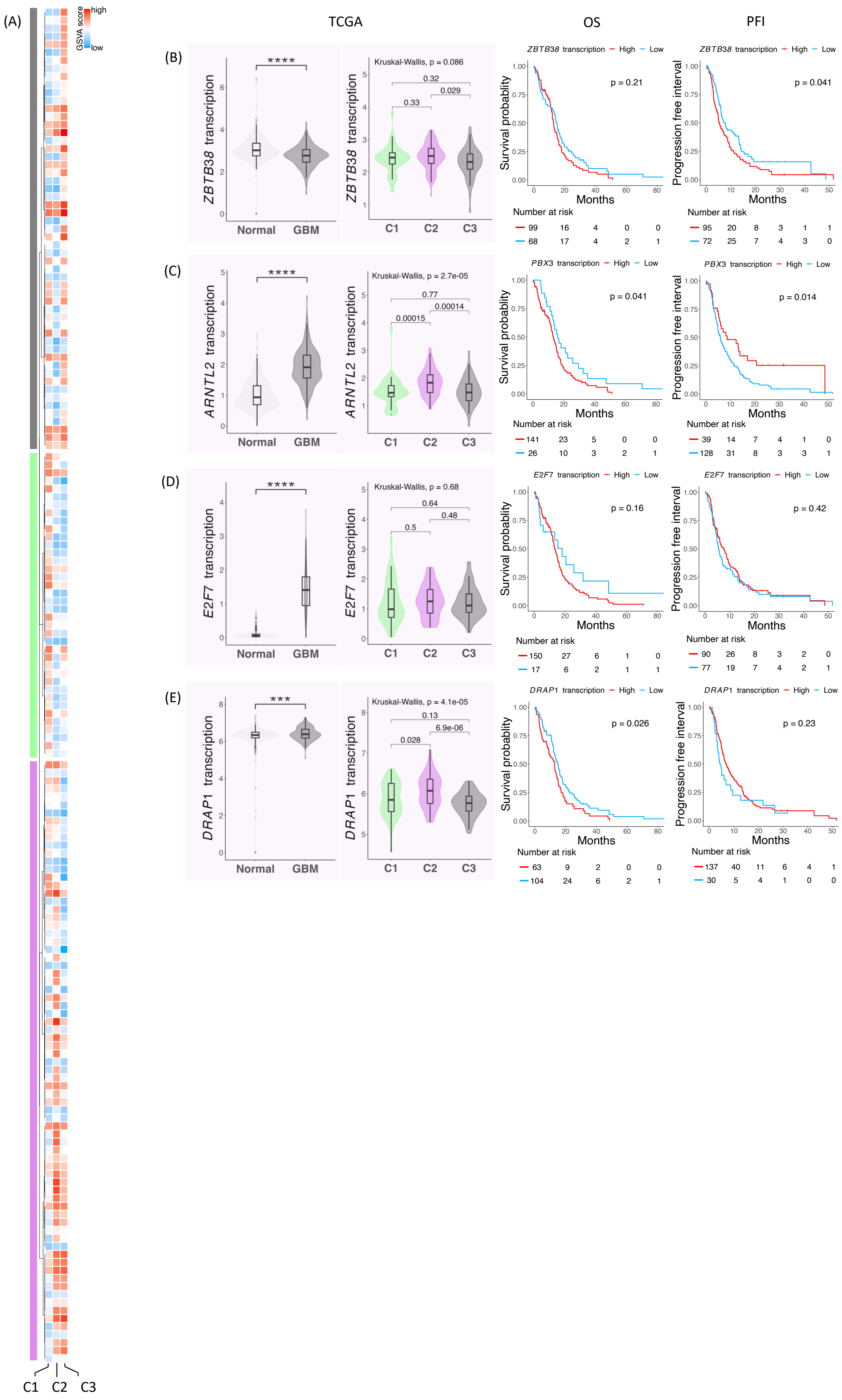

Supplement: Supplementary Figure 1 — (A) Multiple clustering algorithms underscore the robustness of antioxidative network-based classification. Expression heatmap and hierarchical clustering of antioxidative signature; (B–J) Consensus clustering with multiple k values. [file Image_1.pdf]
